# Perception of Survivorship Needs Among Breast Cancer Patients in Trinidad and Tobago

**DOI:** 10.7759/cureus.34394

**Published:** 2023-01-30

**Authors:** Kristy Samaroo, Amalia Hosein, Jameel Ali

**Affiliations:** 1 Biomedical Engineering, The University of Trinidad & Tobago, Port of Spain, TTO; 2 Surgery, University of Toronto, Toronto, CAN; 3 Breast Surgical Oncology, St. James Medical Complex, Port of Spain, TTO

**Keywords:** quality of life (qol), survivorship care, heath related quality of life, survivorship needs, cancer survivorship, breast cancer

## Abstract

The perception of survivorship among the cancer communities of the Caribbean is largely unknown. This study focused on determining the perception and interest in cancer survivorship among breast cancer (BC) patients in Trinidad and Tobago, as a preliminary, to introducing a pilot survivorship program and evaluating its impact on this patient population. Participants were given a questionnaire to determine needs, expectations and interest in survivorship care. Baseline measurable outcomes reported in this article include the following: 1. Participants’ satisfaction with their medical care follow-up plan (if any), 2. Participants’ satisfaction with the amount of information provided by healthcare providers, and 3. Participants’ satisfaction with their physician’s overall concern about their well-being, ranked on a 5-point Likert scale. Participants also reported on the advice/ guidelines provided by their physicians, after surgery and/or on completion of treatment, what they did to cope with BC, and their expectations of what could have been done to improve the quality of care received. A second questionnaire was then used to measure the level of interest in participating in a Cancer Survivorship Program (CSP) with components such as: nutrition, psychosocial development, spiritual well-being, and yoga and mindfulness. The level of interest was ranked by participants on a 5-point Likert scale. Fifteen themes emerged from participants’ responses to the first questionnaire. Nutrition stood out as the module of highest interest, followed by psychosocial development among BC patients.

## Introduction

Survivorship is a term used interchangeably with the term survival, but the definitions are quite distinct. Generally, survival after cancer is described as a function of time. But, integrating quality of life with time, now introduces the concept of survivorship. Survivorship, as defined by the National Cancer Institute [1] focuses on the health and well-being of a person with cancer from the time of diagnosis until the end of life, which includes the physical, mental, emotional, social, and financial effects of cancer that begin at diagnosis and continue through treatment and beyond. The survivorship journey also includes issues related to follow-up care/surveillance, late effects of treatment, cancer recurrence, second cancers, and quality of life [1]. Breast cancer (BC) survivors may be at risk for increased rates of emotional distress and poorer quality of life (QoL), as many of them develop long-term health issues [2, 3]. BC survivorship has only recently earned formal recognition as a research discipline as many adverse health outcomes have been associated with BC and BC therapy. These include anxiety and depression, bone loss and fracture, cognitive decline, venous thromboembolism, stroke, cardiovascular disease and congestive heart failure, climacteric symptoms and sexual dysfunction, neuropathy, pulmonary dysfunction, fatigue, vision problems, thyroid dysfunction, and persistent pain [4].

What is evidently lacking in the literature on cancer survival is a focus on the self-reported needs of cancer patients. This study presents the needs and expectations of survivorship care support among BC survivors, in addition to their self-coping strategies, as described by a cohort of breast cancer survivors in Trinidad (T&T). We also measured patients’ interest in participating in a Cancer Survivorship Program (CSP) oriented towards improved knowledge and management of their health. Interest in educational modules, specifically nutrition, psychosocial well-being, spiritual well-being, and yoga and mindfulness were also evaluated.

## Materials and methods

Participants

A prospective observational study was conducted. The participants were female patients between the ages of 18-69 years with resected breast cancer between December 2020 and June 2021, at the St. James Medical Complex (SJMC) in Trinidad and Tobago. Out of 129 eligible BC patients treated at SJMC, 60 participants agreed to participate in this study. Participation was voluntary, but written consent was mandatory in order to participate. They were each given approximately two weeks to complete a self-administered survivorship questionnaire. Patient demographics were collected from their clinical charts. Approval for this study was obtained from the Research Ethics Committee at the North-West Regional Health Authority, governing the St. James Medical Complex, and the Research Ethics Committee of The University of Trinidad & Tobago.

Measurement instruments

Two questionnaires were designed to collect relevant baseline information from the sample population and to determine needs, expectations and interest in survivorship care. The participants’ responses described their current survivorship experiences. Baseline measurable outcomes reported in this article include participants’: 1. Knowledge about available support services, 2. Satisfaction with their medical care follow-up plan (MCFP) (if any), 3. Satisfaction with the amount of information provided by healthcare providers, and 4. Satisfaction with their physician’s overall concern about their well-being, ranked on a 5-point Likert scale. Participants also reported on the advice/guidelines provided by their physicians after surgery/completion of treatment, what they did to cope with BC and their expectations of what could have been done to improve the quality of care (QoC) received. Participants were then exposed to the concept of survivorship and the delivery of a CSP using a didactic model via virtual sessions conducted by the PI and research team. A second questionnaire was then used to measure the level of interest in participating in a CSP with components such as: nutrition, psychosocial development, spiritual well-being, and yoga and mindfulness. The level of interest was ranked by participants on a 5-point Likert scale.

Statistical analysis

The responses from the 5-point Likert scale were used to compare higher levels of interest (4, interested or 5, very interested) with less interest (3 or less, ranking from neutral, fairly interested to not interested respectively). Univariate tests of association between participants’ demographic and clinical parameters were performed using Welch t-test and Chi-squared tests. For the three qualitative questions asked, participants' responses were manually coded in Microsoft Word (Microsoft® Corp., Redmond, WA) using an inductive approach [5]. They were broadly coded under three main perception themes: how they felt about advice given during treatment, what could have been done to improve the level of care received and what they did to cope with BC.

## Results

Sample description

Demographic Information

The demographic data is reported in Table 1 below. Eleven demographic parameters were analysed to determine their association with interest levels for the interventions, and their current perception and satisfaction of care. Most patients were above the age of 50 (n=51, 85%), with a notable proportion (n=15, 15%) less than 50 years old. All participants identified with a spiritual or religious belief system - 72% identifying with Christianity and the remaining 28% as either Hindu, Muslim, or some other religious belief system. Twenty-five participants (42%) self-identified as being of South Asian ancestry (Indo-Caribbean), with 33% (n=20) identifying with African ancestry (Afro-Caribbean), 23% (n=14) identifying with mixed ancestry and 2% (n=1) identifying with Caucasian ancestry. Most participants were married (58%) and the majority lived with at least one other person in their household (63%). The number of unemployed persons was the same as the number of retirees, making up just over half the group (57%). All participants had some form of educational background with 85% minimally earning secondary school qualifications. The cohort showed markedly high BMI, both at diagnosis and at the time of the study respectively, with most persons being either overweight (43%, 48%) or obese (37%, 25%). There was a 12% reduction in obesity and a 5% increase in the healthy weight BMI, since diagnosis, among the group. From the sample, 73% breastfed their infants, 67% had at least two children and 22% had a history of miscarriage.

**Table 1:** Demographic Information for Participant Cohort

Variable	Percentage or Mean ± SD
Age (Mean ± SD)	55 ± 10
Age (groups, %)	
25-30	2
31-35	3
36-40	7
41-45	3
46-50	20
51-55	15
56-60	10
61-65	27
65-69	13
Age at diagnosis (Mean ± SD)	53 ± 10
Religion	
Christian	72
Muslim	8
Hindu	13
Other	7
Marital Status	
Single	22
Married or Partnered	58
Separated or Divorced	15
Widowed 5	
Living Situation	
Alone	37
Not alone	63
Ethnicity	
Afro-Caribbean	33
Indo-Caribbean	42
Mixed	23
Other	2
Employment Status	
Employed	43
Unemployed	28
Retired	28
Education	
Primary	15
Secondary	40
Tertiary	28
Postgraduate	15
Unknown	2
BMI (current)	
Underweight (<18.5)	2
Healthy weight (18.5-24.9)	25
Overweight (25-29.9)	48
Obese (>30)	25
BMI (at diagnosis)	
Underweight (<18.5)	nil
Healthy weight (18.5-24.9)	20
Overweight (25-29.9)	43
Obese (>30)	37
History of Miscarriage	22
Number of Children (Parity)	
Nil	15
1	18
2	43
>2	23
Breastfed	73

Clinical Characteristics

Eleven clinical parameters, defined in Table 2 below, were used to better understand their association with participants' interest in a cancer survivorship program and its respective elements. These parameters were also used to determine their association with participants’ current perception of survivorship and their degree of satisfaction with received care. The clinical data recorded in Table 2 shows that participants were predominantly diagnosed with invasive ductal carcinoma (98%) of the breast, which is consistent with regional and international statistics. Interestingly, 80% of cases were self-detected, with the highest incidence diagnosed at an early stage i.e., 62% at Stages I to II, whereas 25% were diagnosed at Stage III and 13% at Stage IV. More than half of the group (58%) has a family history of cancer. Most of the participants, 42% of the study, had been diagnosed within less than a year, followed by the remaining 40% between 12 to 18 months and less than one-fifth (18%) diagnosed earlier. Two of the participants (3%) died due to disease progression (metastasis) since the study was conducted, and four participants (7%) were receiving ongoing treatment (either chemotherapy or radiation). The majority of the group was in remission (78%) and five persons (8%) were cancer-free. Eight participants (13%) experienced recurrence, which is a relatively high statistic, considering the time since the first diagnosis among the group (≥ 24 months). Single mastectomy (43%) was the most common surgical procedure for resection of BC, followed by single lumpectomy (wide local excision) (40%). Only one person had a bilateral mastectomy. Chemotherapy and radiation was the primary line of adjuvant therapy (52%), followed by chemotherapy only (25%) and radiation only (13%), while 10% of participants were treated using only hormone or immune-targeted therapy.

**Table 2:** Clinical Characteristics of Participants

Variable	Percentage
Type of Breast Cancer	
Invasive Ductal Carcinoma	98
Invasive Lobular Carcinoma	2
Method of Detection	
Self	80
Other	20
Most Advanced Stage of Breast Cancer	
Stage I	23
Stage II	38
Stage III	25
Stage IV	13
Family History of Cancer	58
Time Since First Diagnosis (months)	
6 to 12	42
13 to 18	40
19 to 24	18
Disease Outcome	
Still in treatment (ongoing)	7
Well	78
Recovered	8
Died	3
Recurrence	13
Cancer-Free Survival Period (years)	
Nil (either still in treatment or died)	33
<1	27
1 to 2	38
>2	2
Type of Surgery	
Single Mastectomy	43
Single Lumpectomy	40
Mastectomy and Lumpectomy	12
Bilateral Mastectomy	2
Bilateral Lumpectomy	3
Cancer Treatment	
Chemotherapy	25
Radiotherapy	13
Adjuvant (chemo + radiation)	52
Other	10

Needs, expectations and satisfaction with care

From the analysis of participants’ responses on (1) what guidelines were provided during their care, (2) what could have been done to improve QoC, and (3) what they did to cope, 15 themes were identified (Table 3).

**Table 3:** Themes Identified from Participants' Responses on Survivorship Needs and Expectations

What advice/guidelines have your providers given you during/after your treatment?	No. of Participants
None	39
Medical-care follow-up plan (Surveillance)	21
Nutritional	5
Stress management	9
What do you think your providers could have done to improve the quality of care you received?	
Providing information	23
Medical system and treatment	16
Support services	9
Acquiescence	20
*How did you cope with your BC journey? Describe some of the things you did to help you get through.*	
Support	21
Attitude	13
Acquiring knowledge	14
Activities	18
Avoidance	3
Spiritual	48
Nutrition	11

Participants' Expectations on How Healthcare Providers Could Have Improved Quality of Care

Four main themes were evident from the qualitative data analysis on BC patients’ expectations on what providers could have done to improve QoC. Participants identified the need for improvement vis-à-vis the provision of information, medical structures and systems, in addition to the need for support services, whilst one-third of participants found that their care was satisfactory.

Providing information: Among participants, the prevailing theme was the need for information. Participants identified wanting to know more about their cancer in general, early detection warning signs, their diagnosis, treatment and side effects, crucial information related to care management (e.g., best practices, what to avoid), aftercare, surveillance, nutrition and coping mechanisms for themselves and caregivers. This is reinforced in the following quotes, taken verbatim, from their questionnaires:

“I would like more information concerning my cancer situation and a general knowledge about cancer. They should be more informative.” (CSP007, age 69, Stage II, also hypertensive)

“I would have liked an information booklet to take with me, as I was already overwhelmed with the diagnosis, and would have liked to be able to read the info once I got home, plus, have it for reference AND also include the places and services that I would need for my aftercare.” (CSP004, age 62, Stage II-A, experienced Grade I lymphedema)

“The providers could have facilitators conducting in-house talks or have videos shown regarding coping strategies, health care tips, and how to help our family cope while we await our turn to be attended to by the doctor” (CSP018, age 48, Stage unknown, also has Lupus)

“Was not told what I can eat or not. Did not know that BP and blood should not be taken from on the side that you had surgery. No one at the hospital told me.” (CSP038, age 55, Stage II, experienced seroma formation and lymphoedema)

Medical systems and treatment: Sixteen participants indicated that their QoC could have been better with improvements in wait time, the atmosphere at the facility, the availability of medication and follow-up care, clerical services for appointments and compassion and support by medical providers. This can be seen in the subsequent quotes taken directly from the participants:

“More efficient service at the clinics that don’t result in such long days for patients.” (CSP043, age 40, Stage II-A, also has hyperthyroidism)

“Process was too long to receive radiation. Medication took long to receive which caused some anxiety.” (CSP003, age 67, Stage II, also hypertensive)

“Provide information and be willing to answer questions that patients may have. If medication is not available, patients should be given an appointment as soon as it becomes available. Long waits are certainly unacceptable. Persons are ill.” (CSP037, age 49, Stage III, also has a heart murmur)

“I guess in terms of speaking more nicer and giving more encouragement, because going through that is scary and we at least want to hear something comforting, especially if it’s from the doctors who diagnose us.” (CSP048, age 46, Stage III, no recorded comorbidities)

Support services: Some participants indicated the need for counselling and stress management services as a means of coping. This was corroborated in the responses below:

“They need a whole new plan. A system where qualified persons are assigned to different areas such as nutrition, coping skills, counselling, etc. so the burden is not on one person alone. You also need people with understanding and who are caring.” (CSP050, age 51, Stage II, also has asthma and glaucoma)

“Interact more, provide counselling services as stress is a leading cause of cancer.” (CSP056, age 52, Stage II, no recorded comorbidities)

Acquiescence: This theme was revealing because one-third of participants indicated their satisfaction with their providers’ care during their survivorship journey. The reflective question demonstrates that some participants are resigned to the systems and structures in place. For example:

“I believe they are doing the best they can, with the tools available to them.” (CSP031, age 54, Stage IV- metastatic BC, also hypertensive)

“I think the quality of care was fair enough, so I have no complaints.” (CSP033, age 35, Stage I, no recorded comorbidities)

“At present, the care is good because I am not experiencing any ill health.” (CSP029, age 68, Stage II, also hypertensive)

Participants' Description of Advice and/or Guidelines Provided by Their Physicians During Treatment

When looking at the participants’ experiences with provided advice and guidelines during their care, four main themes became apparent. Provision of a medical care follow-up plan (MCFP), nutritional advice, stress management tools, with the concerning dominating theme of no guidelines provided. These themes are now expounded further below.

No advice or guidelines provided: Alarmingly, the predominant theme, as reported by 65% of participants, was that no guidelines were provided by their physician or medical team. Participants reported feeling unsupported and having to enquire and search for available services (e.g., counselling and nutrition). In addition, participants indicated that guidelines and coping mechanisms were provided by other BC patients compared to anything being proffered by their health providers. This theme is demonstrated in the following examples taken verbatim from the questionnaires.

“None. A little more informative and follow-up support.” (CSP040, age 69, Stage I, no recorded comorbidities)

“No advice. This had me very concern.” (CSP046, age 56, Stage I, also hypertensive)

“No one spoke about counselling. I had to enquire for myself. I felt unsupported.” (CSP050, age 51, Stage II, also has asthma and glaucoma)

Medical-care follow-up plan (MCFP): Most participants who reported on received guidelines indicated that it was specific to their MCFP, which primarily focused on cancer surveillance. It is interesting that not everyone had an MCFP, since this is an essential part of cancer treatment. Some of the most specific responses on this theme are as follows:

“I have three follow-up visits for cancer surveillance which include periodic mammograms, scans, and blood work. No advice was given to me regarding psychological interventions and coping strategies.” (CSP006, age 53, Stage I-B, no recorded comorbidities)

“Follow-up visits have been scheduled every 6 months e.g., mammograms and breast ultrasounds.” (CSP013, age 61, Stage II, no recorded comorbidities)

“I have to continue with my mammograms for my other breast and be aware of any abnormalities that may develop.” (CSP029, age 68, Stage II, also hypertensive).

Stress management: Stress management advice was obscurely reported by the participants and included suggestions for rest and relaxation, maintaining a positive attitude, having a spiritual focus, and staying healthy and fit. It is important to note that this category of advice was delivered in a non-formal varied format. For example:

“To stay positive, even though I am in remission, that as scary as it is as there is a chance it can come back, but I’m keeping strong and praying to God for the best.” (CSP048, age 46, Stage III, no recorded comorbidities)

“Maintain my positive attitude. Ensure that I get enough rest daily. Maintain my spiritual focus.” (CSP028, age 68, Stage I, no recorded comorbidities)

“Keeping healthy and fit, staying strong and positive.” (CSP047, age 45, Stage IV-metastatic BC, no recorded comorbidities)

Nutritional: The advice and guidelines on nutrition were very vague and unstructured. Only five participants reported to have been advised on nutrition during their survivorship journey. For example:

“I was advised to limit my meat intake.” (CSP013, age 61, Stage II, no recorded comorbidities)

“Diet changes and cancer surveillance.” (CSP014, age 61, Stage III-B, also hypertensive, has arthritis)

“Just to eat everything in moderation.” (CSP039, age 62, Stage II, hypertensive with high cholesterol)

Mechanisms Disclosed by Participants to Help Them to Cope With Breast Cancer

This section shows what mechanisms the participants used to cope with BC during their survivorship journey. Seven major themes emerged. Participants discussed: reliance on social support systems, spiritual beliefs/ practices and being proactive about acquiring knowledge, nutritional modifications, avoidance of negative triggers, in addition to, adopting an attitude of fortitude.

Spiritual beliefs/practices: The most evident coping mechanism reported by participants was their faith and faith-based practices such as prayer, meditation, and devotion. As stated literatim,

“It was tough there were times when I wanted to give up and not continue with chemo because of how it made me feel. The pain was agonizing and not something anyone wants to experience. But through it all, I tried to stay strong and prayed all the time, and God helped me get through it.” (CSP048, age 46, Stage III, no recorded comorbidities)

“I prayed a whole lot. I also had support from my Pastor and family. Strong faith in God and his word is what gave me strength during this journey.” (CSP013, age 61, Stage II, no recorded comorbidities)

“Spending time with the Lord was a tremendous help. Listening to online services, reading my bible and studying the Word of God.” (CSP024, age 64, Stage III-C, also hypertensive)

“My attitude to this diagnosis is very clear, my attitude is that of a winner. My spiritual beliefs have bolstered my faith and by His stripes I am healed. In addition, I always dress up for my appointments because if you look good you feel better. I also do positive affirmations I will live and declare the works of God.” (CSP058, age 48, Stage IV-metastatic BC, no recorded comorbidities)

“I have definitely become more spiritual; could not get through a second of the day without talking to my Maker.” (CSP031, age 54, Stage IV- metastatic BC, also hypertensive)

Social support systems: The second leading coping mechanism described among participants was their reliance on the support of family and friends, including other BC patients. Participants also depended on the support of their medical team to help manage their fears and anxiety. This is evidenced below.

“I coped really well. I have a really supportive family which is a blessing. I spoke to other patients at the center who shared their stories and I read a lot of survivor stories.” (CSP045, age 61, Stage unknown, also hypertensive and diabetic)

“Initially, I felt overwhelmed but my doctor pacified my fears about dying. He encouraged me that I do not have to die from this disease. The fact that I was diagnosed early enough before it spread helped me to stay encouraged.” (CSP003, age 67, Stage II, also hypertensive)

“I relied on the support of my family, especially my mother and sister who are both cancer survivors.” (CSP035, age 63, Stage I, also hypertensive)

Acquiring knowledge: Self-directed information seeking on their BC condition specifically on diagnosis, treatment/side-effects, aftercare and learning more about their chances of survival, using online resources, including cancer survivors’ blogs/testimonies. These were fundamental methods of management among participants. To illustrate:

“I coped pretty well as I Googled the different Cancer sites and read many patients' blogs.” (CSP004, age 62, Stage II-A, experienced Grade I lymphedema)

“Doing my research on my stage of cancer and only listening to positive journeys of BC survivors.” (CSP008, age 47, Stage II, had a hysterectomy)

Activities: Physical and recreational activities helped to keep participants distracted and pleasantly occupied. Some of those highlighted recreational activities were baking, reading, music, television, outdoor activities - breathing in the fresh air and being reminded about life; while others suggested that their daily routines of housework, continued employment and caring for their families helped them to manage. Some examples include:

“I kept distracted by trying to keep up with my normal routine, as much as I could, when I could. Exercising, gardening, playing games on my phone, binge watching shows online, tv.... the little things help.” (CSP031, age 54, Stage IV- metastatic BC, also hypertensive)

“I’ve found a new love for baking and I have been sewing as well, to occupy my time. I’m also spending time reading and studying the Bible.” (CSP015, age 43, Stage III-A, no recorded comorbidities)

Attitude: Participants indicated that having a positive, resilient attitude was helpful. They also expressed the benefits of accepting their diagnosis and being compliant with their treatment. This is demonstrated in the following statements:

“Have a positive attitude and an open mind to treatment. Have a great desire to live. I never felt sorry for myself. I always try to see the positive side of what some may have seen as bad news. I solicit the advice of medical practitioners and I don’t allow the views of non-medical professionals to alter my thoughts, attitude or lifestyle.” (CSP043 age 40, Stage II-A, also has hyperthyroidism)

“At the beginning, when I became aware of my condition, I was very frightened thinking I would pass on; but as time went on, with a lot of prayers, and with the treatment that was prescribed by the consultant, my feelings became more positive.” (CSP029, age 68, Stage II, also hypertensive).

“I had to accept the fact that I had BC and that I had to go through a treatment plan.” (CSP041, age 50, Stage II, no recorded comorbidities)

“I had to remind myself that my body was not 100% and to slow down (when I started to heal). Allow my family to help me to do things. I had to stop being so self-conscious.” (CSP039, age 62, Stage II, hypertensive with high cholesterol)

Avoidance: As a mechanism, avoidance can be related to the preceding theme of attitude, as participants described their determination, in ascribing to specific behavioural modifications, to improve survivorship. Three persons, quoted below, spoke about their need to avoid negative situations as a way to cope with their BC.

“I solicit the advice of medical practitioners and I don’t allow the views of non-medical professionals to alter my thoughts, attitude or lifestyle.” (CSP043, age 40, Stage II-A, also has hyperthyroidism)

“I also did not tell too many people of my situation, as their reaction was taking me back to how I felt, when I first got the diagnosis.” (CSP039 age 62, Stage II, hypertensive with high cholesterol)

“I stayed away from negative situations and people.” (CSP056, age 52, Stage II, no recorded comorbidities)

Nutrition: Nutritional changes were also mentioned by just under 20% of participants, as a way to manage their BC. While most of these participants generally stated “diet changes or eating well”, two participants specified in more detail as follows:

“I found joy in preparing healthy foods and used my time to learn about what causes cancer, how I can selfheal and nourish my body and prevent a reoccurrence.” (CSP056, age 52, Stage II, no recorded comorbidities)

“I became more aware of what I ate and eliminated some foods from my diet and get more disciplined with my daily exercise.” (CSP029, age 68, Stage II, also hypertensive)

Satisfaction With Survivorship Care

Participants’ responses gave us an idea of how satisfied BC patients were with their standard of cancer care as shown in Table 4 below.

**Table 4:** Satisfaction with Cancer Care among Breast Cancer Participants

Parameter	Medical Care Follow-up Plan (MCFP)	Information Provided by Physicians	Physicians’ Concern about Overall Well-being
% Satisfaction	43	72	60

Table 4 reveals that less than half of the participants (43%) were satisfied with their MCFP. Generally, the fundamental focus of survivorship care plans includes surveillance for cancer recurrence, long-term side effects and late effects of treatment. This recent focus stems from the prominent 2006 report, From Cancer Patient to Cancer Survivor: Lost in Transition, by The Institute of Medicine [6], which has since gained further attention from other researchers, suggesting more emphasis on patient education about long-term sequelae of this disease and its treatment [7]. It is therefore disturbing that more than half of our participants found their MCFP to be unsatisfactory, since it is such an essential part of recommended cancer care. Though most participants (72%) were satisfied with the amount of information provided by physicians (Table 4); contrary to what was reported earlier, 23 participants (38%) indicated that they desired more information when asked what could have been done by providers to improve the quality of care received (Table 3). Interestingly, Chua et al. [8] found that almost all surveyed cancer patients wanted information about the disease, tests, investigations, treatment, side-effects, sexuality, psychosocial support, and financial matters, and signified that patient satisfaction with the information provided is one important indicator of the quality of care delivered. These results indicate the need for informational booklets that can educate patients about their condition from the inception of diagnosis. To properly facilitate the emerging trend of integrated, patient-centered care, Davis et al. [9] suggested that patient education and meeting patients’ information needs must be considered as an essential aspect. Chua et al. [8] contend patient education that corresponds to patients’ needs, values, wishes and psychological circumstances is likely to be most effective [10]. It was concerning to find that only 60% of participants were satisfied with their physicians’ concerns about their overall well-being (Table 4). Patient dissatisfaction with physician support during care is not isolated to this region [11, 12]. Coronado et al. believe the impact of the patient-provider relationship can affect the survivorship experience, since patients and caregivers who perceive care interactions as supportive, will tend to trust their recommendations for treatment [13]. Conversely, negative provider behaviour can discourage patients from participating in discussions about their care [13]. This view is also supported by Mollica et al. in their scoping review of patient experiences of cancer care [14]. Gupta et al. found that completely satisfied BC patients had a significantly lower risk of mortality compared to those not completely satisfied (HR = 0.62; 95% CI 0.50-0.76; p < 0.001) [15].

Interest among participants in a survivorship intervention

This section measures interest in a Cancer Survivorship Program (CSP), a didactic model with a nutritional module, a psychosocial module, a spiritual module and a yoga mindfulness module (Table 5). Data analysis was undertaken based on levels of high and low interest and whether they were associated with demographic and clinical parameters.

**Table 5:** Interest among Participants in a Cancer Survivorship Program (CSP)

Parameter	CSP	Didactic Model	Nutrition Module	Psychosocial Module	Spiritual Module	Yoga & Mindfulness Module
% Interest	90	87	97	90	68	67

Unsurprisingly, 90% of the participants showed high interest in participating in a CSP (Table 5). Only two participants were neutral, and none indicated no interest. However, we noticed that persons who had just started radiotherapy were significantly (p=0.038) less interested compared to participants who completed more than 10 radiotherapy treatments (Table 2). This is understandable since it is well known that cancer-related fatigue is reported by a substantial majority of breast cancer patients during their initial radiation treatment [16]. This, often described as a nebulous symptom, can account for the lower interest seen in this group.

There was 87% interest in a didactic model of a CSP among the group of participants. Participants were exposed to the concept of a didactic model as being one that is educational, designed to achieve lifelong coping skills, by applying the principles of effective teaching and learning, referring to Bloom’s Taxonomy of the Cognitive Domain [17], Krathwohl’s Taxonomy of the Affective Domain [18] and the Taxonomy of Psychomotor Skills development [19]. All three taxonomies were used to enhance retention of skills, attitudes and cognitive elements of learning.

Overall, 97% of the participants were interested in a nutritional module, which was the highest recorded interest level, among participants for any module/parameter measured. This heightened interest could be due to recent research trending in nutrition and healthy living in the fight against cancer. In 2018, the World Cancer Research Fund (WCRF) and American Institute for Cancer Research (AICR) published the Third Expert Report entitled, Diet, Nutrition, Physical Activity and Cancer: A Global Perspective that extensively covers the role of nutrition in improving survival outcomes as well as its possible role towards cancer prevention [20].

As expected, there was also high interest in the psychosocial module among the majority (90%) of participants. Depression and anxiety are often neglected areas of management in cancer patients, even though depression is a significant complication of cancer with higher occurrence than in the general population [21]. Pinquart and Duberstein suggested that a depression diagnosis, coupled with higher levels of depressive symptoms predicted elevated mortality in cancer patients [22]. As we can infer from the participants’ very own words, a cancer diagnosis comes with anxiety and requires proper counselling and stress management in order to cope. This need is further endorsed by high-interest levels. Older participants (56 ± 10) showed a significantly (p=0.03) higher interest in the psychosocial module compared to relatively younger participants (45 ± 9) who were only fairly interested. Similarly, participants who were first diagnosed at an older age (55 ± 10) were significantly (p=0.036) more interested in psychosocial intervention compared to those diagnosed at a younger age (43.5 ± 9). There was also a significant (p=0.04) interest in this module among 75% of participants with metastatic breast cancer. Interestingly, among the two participants who died due to disease progression, one was interested in psychosocial development while the other was neutral.

The lowest recorded interest, shown among participants, was for the yoga and mindfulness (67%) and spiritual (68%) modules respectively. Over a quarter of participants (25.6%) who identified with the Christian faith, as well as 75% of Jehovah Witnesses, showed less interest in yoga and mindfulness. Surprisingly, 62.5% of Hindus were also less interested in yoga and mindfulness, whereas 80% of Muslims showed interest in this module. Three persons were not interested in the yoga and mindfulness module. These results were most likely due to stigmatization and conflicting religious and spiritual beliefs about yoga. However, many studies have proven the tremendous benefits of yoga in improving rates of survivorship. In their review of evidence-based research, Agarwal and Maroko-Afek revealed that yoga improved physical and psychological symptoms, quality of life, and markers of immunity of the patients, providing strong support for yoga’s integration into conventional cancer care [23].

The lower levels of interest in the spiritual module can be attributed to the indication that all participants had already ascribed to a particular religious belief that seemed to work well for them. As such, they may not need further spiritual support. Spirituality in Cancer Care (PDQ®)-Patient Version, published by the National Cancer Institute, informs that many patients with cancer rely on spiritual or religious beliefs and practices to help them cope with their disease, coined “spiritual coping” [24]. A meta-analysis conducted by Jim et al. found that religion (religious beliefs/practice) and spirituality (a connection to a source larger than oneself and feelings of transcendence) were associated with overall physical health and well-being, functional well-being, and physical symptoms [25].

## Discussion

This aspect of the project used a combination of open-ended questions on expected, perceived, and current survivorship care among BC participants from Trinidad & Tobago (T&T). From this research, we were able to attain insights into the needs of participants during their BC journey. The need for information and knowledge was identified as an overarching theme. Generally, participants expected more information (38%) specifically at the point of diagnosis, to prepare them for their imminent BC journey. However, most of the information was acquired from their own research and other patients’ advice (23%). The participants revealed that more information during their journey would have improved their treatment regimens, nutritional choices, managing side effects of treatment, and more. It has been recognized that patient education and knowledge, at diagnosis and onwards, can improve patient outcomes [26].

The need for support services such as mental health, stress management and nutrition, was another critical theme emerging from this study. Following a cancer diagnosis, just nine participants (15%) expected to receive counselling (psychosocial support) from their care providers, while one-third of the participants were resigned and had no further expectations. It is well recognized in the literature on psycho-oncology that psychological interventions, not only at the time of a cancer diagnosis but throughout survivorship, can improve outcomes [27]. It is well known that several gaps exist in primary health care and health systems in the Latin American and Caribbean region, adversely affecting the quality of care [28]. Hence, due to the construct of healthcare systems and structures in the Caribbean, persons do tend to expect assistance more than their standard clinical treatment. This reemphasizes the need for psychosocial support services, to be included as part of the necessary standard of care, throughout cancer survivorship. The need for stress management techniques was also identified as a means to improve QoC. Participants indicated that a cancer diagnosis caused stress and anxiety and expected measures to help them cope. Advice on strategies to manage stress was informally given by physicians in an unstructured manner, dependent on the treating physician and patient relationship. Molinaro et al. purport the impact of psycho-oncology in reducing distress and depression in cancer patients [27]. Techniques such as relaxation and meditation, as well as, physical and recreational activities were discovered by participants for stress management. This highlights the gap between the need for coping measures and the need for the provision of techniques by providers, in a uniform structured manner with diverse approaches to stress management as part of the standard of care.

Participants identified the need for nutritional advice during their survivorship journey. Participants expected that guidelines and dietary advice would have featured more prominently in their treatment plan. Nutritional guidelines should be a part of standard cancer care, considering the substantial impact of nutrition on patients’ survivorship rates [29].

It is important to note that the practice of faith-based religion was largely prominent as the major coping mechanism among the majority of participants (82%) during their BC journey. This implies that a spiritual belief system improved coping with cancer, which is strongly supported in the literature. Spirituality is an effective adaptive coping strategy and a predictor of a better quality of life in cancer patients [30].

This study acknowledges effective patient-directed coping strategies such as a resilient attitude, positive affirmations, adherence to clinical advice/treatment plans and avoidance of negative triggers (such as circumstances that caused them to relive their diagnosis). There were patient-oriented coping tools that stood out, such as religion, attitude and knowledge-seeking. Those were keystone factors in participants’ survivorship journey. Most participants described adopting these practices to be able to endure the hardships of their condition.

Additionally, we used 5-point Likert scales to determine interest in a Cancer Survivorship Program which included interventions such as a didactic module, a nutritional module, a psychosocial module, a spiritual module, and a yoga and mindfulness module. The results convincingly showed that there was a high interest in a CSP (90%) among cancer patients. However, due to substantial interest in a CSP, significance among the various parameters that were tested showed little variation. Participants showed higher interest in nutrition and psychosocial modules compared to the spiritual and yoga and mindfulness modules. Generally, as analysed, there was considerable interest (≥60%) in all areas of the CSP.

Limitations

The responses were all self-reported by participants in the study. Self-reporting is associated with their own biases [31], however, this was the best approach within the local system and structure to obtain perception information. The research questions may have influenced participants to provide desirable answers to the researchers as participants enrolled in this study based on their agreement to participate in a pilot CSP. The administering of an online questionnaire limited participants with internet access, a comfort with technology and typing skills.

In addition, the nature of perception reflects the participants' reality based on their background such as level of education and individual cultural differences and may not be the actual situation as is reported in this paper. The participants were all patients treated at one primary institution in T&T. However, the study site is the main cancer treatment center in Trinidad and Tobago and will capture a large proportion of BC patients. Lastly, this research was done during the COVID-19 pandemic whilst transitioning to lockdown which may have allowed for less access to public health facilities and perceptions may not be representative of the actual public health setting for past or future patients. This merits continued prospective insight into cancer care needs to better validate representative BC perceptions.

## Conclusions

This study demonstrated that BC patients have specific information needs that require attention by the primary cancer treatment center in T&T. This can be effectively achieved through the dissemination of informational booklets, at the point of diagnosis, to educate patients on their cancer to help manage expectations. Further to this, participants desire improvements in the medical system and structures, particularly in the lengthy wait time in the clinic, and at follow-up services. Other improvements mentioned were better support from the medical team and the need for support services such as counselling to manage stress/anxiety resulting from a BC diagnosis, as well as advice on nutrition to improve health management. Participants generally described the advice or guidelines provided by physicians as vague and non-standard, which could be attributed to patient-provider relationships and/or personal understanding. Notable was that the majority of patients indicated that no advice or guidelines were given specifically related to cancer surveillance for recurrence/secondary cancers, late effects of treatment, psychological interventions and coping strategies. Some participants suggested that because treatment was ongoing, they may not have yet received advice or guidelines for future management. However, participants who completed treatment also reported that no such advice/guidelines were given. Seven themes emerged from patient-directed coping strategies: spiritual support, knowledge-seeking, activities (hobbies, physical), attitude, avoidance and nutrition. These findings provide evidence for the establishment of a holistic survivorship program encompassing the unmet needs of cancer patients to enhance coping.
